# The Mental Deficiency Act at Work

**Published:** 1918-03-30

**Authors:** 


### THE MENTAL DEFICIENCY ACT AT WORK.

### A Surprising War-Time Achievement.

The Report of the Board of Control for the year 1916 contains some extremely interesting information concerning the working of the Mental Deficiency Act, 1913. The administration of the Act has, of course, been sadly hampered by the consequences of the war—by the dearth of *personnel*, by the scarcity of funds, by the difficulty of providing proper and sufficient accommodation, and by all the difficulties that are felt in every department of life and activity, owing to the diversion of our interests, our man-power and woman-power, our money, and every kind of material to the service of the war. In the circumstances it is surprising that anything at all has been done, and we record with great satisfaction that much has been done, at any rate in certain districts and at certain centres. Altogether, 6,836 persons (3,093 males and 3,743 females) have been dealt with under the Act, by far the greater number having been placed in institutions. A minority of 333 are boarded in approved homes, and a still smaller minority of 136 are merely under guardianship. There are already as many as forty-four institutions certified under the Mental Deficiency Act, and in regular work, and of these only six were previously registered under the Idiots Act, 1886. Of the remainder only seven have been established by local authorities, and the remaining thirty-one have been organised by incorporated bodies, or religious or other societies, or committees of private persons. In spite, therefore, of the enormous demands made by the war, private enterprise and private philanthropy are still active amongst us. Few things could show more conclusively how healthy is the nation at heart.

Eight new institutions were certified in the year under review, and the account of them makes inspiring reading, for it shows how much can be made of even very unpromising human material by the assiduous care of devoted and judicious supervisors, and it shows, what is almost more surprising, a genial, helpful, encouraging attitude on the part of the Commissioners of the Board, very different from the frigid and formal officialdom of the old Commission in Lunacy. Such a report as the following concerning the Western Counties Institution at Starcross would have been impossible under the old Board of Lunacy:—

"The whole place was found in excellent order on both occasions, and it was fully evident that patients generally are carefully tended, busy, happy, and contented. Although many institutions make a feature of school work, manual training, and employment [we should have thought the first two at any rate were two features, but where the intention is so good it is hypercritical to scrutinise the expression too closely], there are few that equal the thoroughness of Starcross in this respect. In the morning schools the children are carefully graded, and 'very few instances were found where [? in which] one pupil was either far in advance or below the average of his class. The classes are small, seldom exceeding fifteen or sixteen.' 'The afternoon classes are devoted to manual work, and are of special interest. It is here that children begin to acquire a facility in the use of their hands that enables them to take part in the various industries for which this institution is famous. The most striking feature of the teaching is the small amount of preliminary finger training given to children; each begins, almost from the first, to do some work of real use to the institution, the finger training being obtained in the process [obviously the proper way, and the only effectual way, to train the children to use their fingers. Formal 'exercises' intended to train the fingers are mere waste of time.] In this regard it is interesting to see at what an early age a boy or girl, under careful supervision, is able to make a suitable article. One class of boys was busy completing an order for eight gross of strawberry baskets, and a class of girls were turning out a large number of dolls' hats, for which there seems to be an unfailing demand. With the exception of the cord trousers worn by the boys, all the clothing for patients, the uniform for staff, and the sheets, blankets, and household linen are woven and made at the institution. The patients also make and repair all their own boots, and have a considerable outside trade in rugs, cocoanut-fibre mats, baskets, lace, and toys. They are now making a large number of cordite bags for the Navy, and have supplied many hundreds of pairs of socks for soldiers. . . . The present shortage of farm labour has given the superintendent a great opportunity. Farmers in the neighbourhood constantly apply for help, and he is able to send an attendant with a batch of from twelve to eighteen boys out to work. The plan has proved most successful, it being found that, under guidance, these boys can manage to do most of the ordinary farm work.

A more encouraging and inspiring report we have never had the pleasure of perusing. It shows results that are positively astonishing considering the unpromising character of the children employed; for it is to be remembered that these children are all mentally deficient, feeble-minded, and imbeciles, though no doubt they are for the most part not gravely defective. And it is to be remembered that many of the children who constitute this busy hive of industry are taken from slums; that they are of the class from which the feeble-minded criminal and prostitute are recruited; that if left in their home surroundings they would be sunk in idleness, immorality, and sloth, and would, many of them, be tenants of the misnamed workhouse, many of them would be in prison or qualifying for prison, and many of the girls would have already have started on a life of prostitution, and would be bringing into the world a brood of children to be as idle and worthless as themselves, and, like themselves, a burden on the State. Such an institution of itself justifies the great expenditure on the Royal Commission, to whose labours the Mental Deficiency Act, 1913, is due.

